# The Role of Lipid Metabolism in COVID-19 Virus Infection and as a Drug Target

**DOI:** 10.3390/ijms21103544

**Published:** 2020-05-17

**Authors:** Mohamed Abu-Farha, Thangavel Alphonse Thanaraj, Mohammad G. Qaddoumi, Anwar Hashem, Jehad Abubaker, Fahd Al-Mulla

**Affiliations:** 1 Department of Biochemistry and Molecular Biology, Dasman Diabetes Institute, 15462 Dasman, Kuwait; mohamed.abufarha@dasmaninstitute.org; 2 Department of Genetic and Bioinformatics, Dasman Diabetes Institute, 15462 Dasman, Kuwait; alphonse.thangavel@dasmaninstitute.org; 3 Pharmacology and Therapeutics Department, Faculty of Pharmacy, Kuwait University, 13110 Kuwait City, Kuwait; mohammad.qaddoumi@dasmaninstitute.org; 4 Department of Medical Microbiology and Parasitology, Faculty of Medicine, King Abdulaziz University, Jeddah 11633, Saudi Arabia; amhashem@kau.edu.sa; 5 Vaccines and Immunotherapy Unit, King Fahd Medical Research Centre, King Abdulaziz University, Jeddah 80205, Saudi Arabia

**Keywords:** coronavirus, COVID-19, SARS-COV-2, lipid metabolism, sphingolipid, endocytosis

## Abstract

The current Coronavirus disease 2019 or COVID-19 pandemic has infected over two million people and resulted in the death of over one hundred thousand people at the time of writing this review. The disease is caused by severe acute respiratory syndrome coronavirus 2 (SARS-CoV-2). Even though multiple vaccines and treatments are under development so far, the disease is only slowing down under extreme social distancing measures that are difficult to maintain. SARS-COV-2 is an enveloped virus that is surrounded by a lipid bilayer. Lipids are fundamental cell components that play various biological roles ranging from being a structural building block to a signaling molecule as well as a central energy store. The role lipids play in viral infection involves the fusion of the viral membrane to the host cell, viral replication, and viral endocytosis and exocytosis. Since lipids play a crucial function in the viral life cycle, we asked whether drugs targeting lipid metabolism, such as statins, can be utilized against SARS-CoV-2 and other viruses. In this review, we discuss the role of lipid metabolism in viral infection as well as the possibility of targeting lipid metabolism to interfere with the viral life cycle.

## 1. Overview of RNA Viruses and a Special Focus on the COVID-19 Virus

The rapidly growing Coronavirus Disease (COVID-19) pandemic represents a serious global challenge [1]. The emergence of SARS-CoV-2 has been manifested as the third revelation of a highly pathogenic coronavirus into the human population after the severe acute respiratory syndrome coronavirus (SARS-CoV) in 2002 and the Middle East respiratory syndrome coronavirus (MERS-CoV) in 2012 [2,3]. Coronaviruses are a family of enveloped viruses with a large single-stranded positive-sense RNA genome, named for their crown-like appearance under the electron microscope [4]. The factors that influence the survival of such viruses on various surfaces depend on several factors such as the viral load, type of surface, suspension medium, humidity, temperature, and others [5]. Following COVID-19 infection, SARS-CoV-2 can penetrate the mucous membranes of the nose, eye, and/or mouth and move on to other vital organs such as the lung. Infections with SARS-CoV-2 range from asymptomatic or mild infections restricted to the upper respiratory tract to severe respiratory syndromes manifested by disseminated spread to the lower airways leading to local inflammation and pneumonia, especially in patients with comorbidities such as diabetes, hypertension, and cardiovascular disease (CVD) [6]. People with diabetes mellitus (D.M.), severe obesity, and hypertension are more prone to be infected and are at a higher risk for complications and mortalities from COVID-19 [7]. The Chinese Centre for Disease Control and Prevention reported increased mortality in individuals with diabetes (2.3% for overall vs. 7.3% in patients with diabetes) from 72,314 cases of COVID-19 [8]. Severe obesity and hypertension are present in 15.5% and 68.4% of D.M. individuals, respectively. While most severe infections affect the elderly above 60 years, children on the contrary seem to be less affected by COVID-19 for reasons yet to be elucidated [9].

Virally infected cells seem to require higher metabolic alterations in order to deal with the high anabolic demands required during viral replication [10,11,12,13]. Virion production requires a rearrangement of the entire biosynthesis apparatus, a process that usually involves major changes in the cellular lipidome [10,11,12,13]. Nonetheless, there are highly exclusive patterns of virus-induced remodeling of host cell metabolic machineries, and the mode of cell manipulation appears to be different between DNA and RNA viruses [12,13]. Recent data suggest that while transcriptional regulation of key metabolic pathways is seen with several DNA viruses [14,15,16], RNA viruses appear to control host-cell metabolism via post-transcriptional regulations [10] to cope with the pace of the corresponding replication cycles.

Lipids play a central role in viral infection, as they represent the structural foundations of cellular and viral membranes [17]. Viruses attack lipid synthesis and signaling to modify host cells to produce lipids for their envelopes [18]. Lipid involvement in membrane fusion, envelopment, and transformation are important for viral replication, and molecules that impact lipids such as cholesterol and sphingolipids could be targeted to selectively impede viral replication [17]. Viruses replicate within the host cell; hence, they must cross the host cellular membrane for entry and exit [17]. Lipids have several roles in viral invasion, as they can act as direct and indirect viral receptors, fusion cofactors, and entry cofactors [18].

Currently, there is no effective vaccine or drug for SARS-CoV-2 except for supportive and empirical medications including non-specific antivirals such as interferons or monoclonal antibodies [19,20]. Convalescent plasma from recovered patients has been utilized with some success, but several clinical studies are ongoing to evaluate its efficacy. More than 50 vaccines based on different platforms are under development with some in phase I trials [21,22,23,24,25,26,27,28]. While understanding the diverse roles of lipids in viral replication has led to the discovery of lipid-active compounds as possible antiviral agents, current compounds mostly lack specificity and are hence excessively toxic. Clearly, there is a need for multiple therapies for COVID-19, and studies have shown that viral entry, release, and consequently replication could possibly be blocked by altering or targeting membrane lipid composition and/or lipid metabolism, depicting new possibilities for antiviral therapies [17].

## 2. Role of Lipids in Viral Metabolism and Membrane Formation

The host lipid biogenesis pathways play crucial roles in controlling virus replication. Lipids can act as direct receptors or entry co-factors for all types of viruses at the cell surface or the endosomes [29,30]. They also play an important role in the formation and function of the viral replication complex, as well as the generation of the energy required for efficient viral replication [11,12,13]. Moreover, lipids can regulate the appropriate cellular distribution of viral proteins, in addition to the assembly, trafficking, and release of viral particles [31,32].

Coronaviruses firstly seize host cell intracellular membranes to create new compartments known as double-membrane vesicles (DMVs) needed for viral genome amplification. A specific phospholipid composition is required by different viruses to form the perfect replicative organelles that are suitable for their replication [33]. DMVs are membranous structures that contain viral proteins and an array of confiscated host factors, which jointly orchestrate an exclusive lipid micro-environment ideal for coronavirus replication [34]. A recent study indicated that cytosolic phospholipase A2α enzyme (cPLA2α), a crucial lipid processing enzyme belonging to the phospholipase A2 superfamily, is critical for DMVs’ formation and coronaviruses’ replication [35]. Accumulation of viral proteins and RNA, as well as creation of infectious virus progeny, were significantly reduced in the presence of cPLA2α inhibitor [35]. Similarly, increased expression of age-dependent phospholipase A2 group IID (PLA2G2D), an enzyme that usually contributes to anti-inflammatory/pro-resolving lipid mediator expression, resulted in worsened outcomes in aged mice infected with SARS-CoV, suggesting that inhibition of such factor could represent a potential therapeutic option [36]. However, to date, the change and tempering effects of specific lipids involved in lipid remodeling upon coronavirus infection stay largely unexplored.

Earlier reports have shown that most RNA viruses such as rhinoviruses show an enhancement of glucose uptake and dependence on both extracellular glucose and glutamine for optimal viral replication [10]. This glucose uptake enhancement and phosphoinositide 3-kinase (PI3K)-regulated glucose transporter (GLUT1) upregulation were found to be PI3K driven and reversible by PI3K inhibitor. Moreover, metabolomic studies revealed increased levels of metabolites associated with glycogenolysis, a process that has not been described so far in the context of viral infections. Lipogenesis and nucleotide synthesis were found to be elevated as well. Lack of both glutamine and glucose impaired high-titer rhinoviruses replication in cells, and the glycolysis inhibitor 2-deoxy-d-glucose (2-DG) effectively repressed viral replication and reversed the rhinoviruses-induced alterations of the host cell metabolome. These findings underscore the potential of metabolic pathways as a target for host-directed antiviral therapy [10].

Citrate, which is the main carbon source for fatty acid (F.A.) or cholesterol synthesis, can usually pass across the mitochondrial membrane and is cleaved into acetyl-CoA that gets carboxylated by acetyl-CoA carboxylase (ACC) to yield malonyl-CoA [37,38,39]. On the other hand, F.A. synthase (FASN) catalyzes the production of palmitic acid (C16:0) from cytosolic acetyl-CoA and malonyl-CoA. The palmitic acid can then be further processed and used in the synthesis of cell membranes, storage in lipid droplets, or the palmitoylation of host and viral proteins. As for sterol biosynthesis, two units of acetyl-CoA are processed to make acetoacetyl-CoA, which then enters the metabolic 3-hydroxy-3-methyl-glutaryl-CoA reductase (HMG-CoA reductase) pathway for cholesterol production. Moreover, F.A.s can be metabolized by catabolic beta-oxidation to yield high amounts of ATP [39,40,41]. Cholesterol and F.A. are essential for viral replication as they constitute the main components of viral membranes. Hence, the metabolism of these lipids has been shown to be required for the replication of various viruses [42]. FASN enzyme is an important player in this process as it controls F.A. synthesis and can regulate the viral replication. Some viruses can even harness this process by increasing the expression of FASN and enhancing their activity [42].

As previously shown, the impact of viral infection on various lipid species has been dramatic. Wu et al. showed that the effect of treatment on SARS patients was long-lived and could significantly impact their serum metabolites. In their study, Wu et al., recruited 25 SARS survivors 12 years after their infection and compared them to healthy controls [43]. They demonstrated that SARS survivors were more susceptible to lung infections, tumors, cardiovascular disorders, and abnormal glucose metabolism compared to controls. More importantly, to this review, SARS survivors had a higher level of phosphatidylinositol and lysophosphatidylinositol compared to uninfected controls. This increase in metabolites was believed to be caused by the high dose of steroid treatment using methylprednisolone [43].

In another study, Nguyen et al. explored changes in the lipidome of primary human epithelial cells infected with rhinoviruses [44]. Post-infection untargeted lipidomics analysis of infected cells showed time-dependent changes in multiple lipid pathways and in the length of fatty acid saturation and class. These pathways implicate multiple lipid modifying enzymes, which were increased upon infection including lysocardiolipin acyltransferase (LCLAT), phosphoinositide phosphatase (PIP), and D.G. kinase amongst others that present good antiviral targets [44]. Other studies have also looked at changes in the lipidome profile after infection with viruses such as Enterovirus A71 and coxsackievirus and found that 47 lipid species/classes such as arachidonic acid, docosahexaenoic acid, and eicosapentaenoic acid were consistently upregulated after infection [45]. These data highlight the increase in lipid metabolism that occurs after viral infection where the virus highjacks and utilizes the host lipid metabolism for their own propagation.

## 3. Viral Internalization: Lipid-Mediated Endocytosis

Endocytosis is the process of internalization of various materials into the cell. It is used to internalize fluids, cellular components as well as various solutes. This happens through the invagination of the plasma membrane and the internalization of various components into cells through membrane vesicles. Viral entry is dependent on the attachment and fusion of viral membrane with plasma membrane through an endocytosis-mediated process [46,47]. Of high relevance to this review and the ongoing COVID-19 pandemic is the role of lipid rafts in viral entry into the host cells. Lipid rafts are sphingolipid-, cholesterol-, and protein-rich microdomains of the cell membrane. Lipid rafts have been found to be important to multiple viruses including SARS-CoV, especially during the early replication stage, although there was no increased angiotensin-converting enzyme 2 (ACE2) localization in such rafts [48]. This could be compensated by the presence of caveolins, clathrins, and dynamin that are important for the endocytosis process and viral entry. This process facilitates the fusion as well as the release of the viral genome into the host cell, where many enveloped viruses, including coronaviruses, take advantage of the low pH inside the endosomes to facilitate the fusion and viral genome release [48].

SARS-CoV-2 belongs to the Betacoronavirus genus in the subgenus Sarbecovirus, which includes SARS-CoV but not MERS-CoV, which belongs to Merbecovirus subgenus [49]. The genome structure of SARS-CoV-2 has a gene order of 5′-replicase ORF1ab-S-envelope(E)-membrane(M)-N-3′, which is shared by other Betacoronaviruses [50]. The Spike (S) protein is made of two functional subunits (S1 and S2) both of which are necessary for virus entry into host cells. While S1 contains the receptor binding domain (RBD) required for viral attachment to host cell receptors, S2 facilitates the fusion of the cell and viral membranes. The RBD of the S protein from SARS-CoV-2 is well-suited for binding to the human ACE2 receptor, which is also used by the SARS-CoV albeit more efficiently in the former virus [50]. For the fusion to take place, the S protein needs to be cleaved by cellular proteases to expose the fusion sequences [51,52]. The amino acid sequence of SARS-CoV-2 S protein, unlike other Betacoronaviruses, contains a characteristic insertion of polybasic cleavage site (residues PRRA) for furin (alias PCSK3) at the junction of S1 and S2 subunits. Furin is a proprotein convertase, which is a serine proteinase. Proprotein convertases are involved in posttranslational processing of the precursors of a vast number of cellular proteins [53]. It was recently reported that SARS-CoV-2 uses the serine protease TMPRSS2 for S protein priming and it is speculated that furin-mediated precleavage at the S1/S2 site in infected cells might promote subsequent TMPRSS2-dependent entry into target cells, as reported for MERS-CoV [54].

## 4. Targeting Lipid Metabolism Pathways

**Figure 1:**
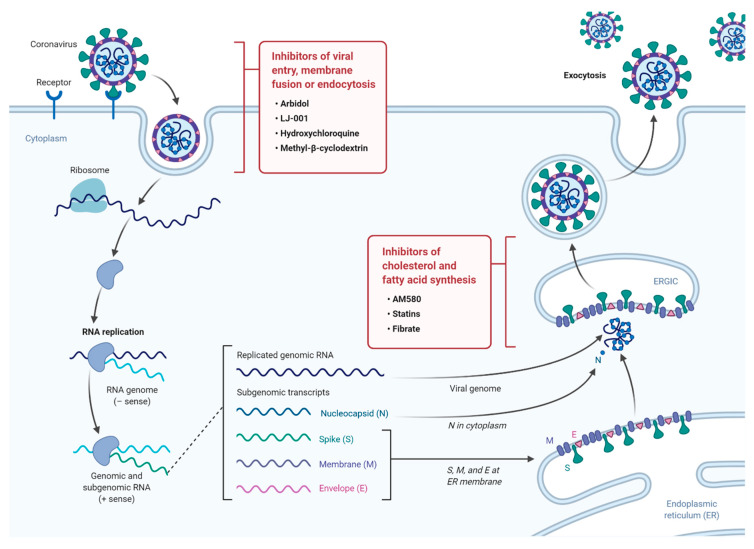
A diagram illustrating the life cycle of SARS-COV2 and potential lipid modifying drugs that can used as broad-spectrum antiviral drugs to inhibit viral entry, membrane fusion, or endocytosis as well as inhibition of fatty acid and cholesterol synthesis.

The continuous emergence of new strains of coronaviruses such as SARS-CoV-2 constitutes a major challenge as global efforts are in a race with time to identify treatments and develop new vaccines. These are generally slow processes that require extensive research and development and ultimately delay the responses to such emerging epidemics. On the other hand, the development of broad-spectrum antiviral drugs could constitute an important first response to such epidemics and enhance the impact of such responses. Pathways that are fundamental to viral attachment and fusion as well as pathways involved in the viral replication and assembly are the foundations for drug discovery. Please see Figure 1 for a summary of current anti-viral drugs that are mentioned in this article [42].

## 5. Targeting Lipid Synthesis Pathways

One important pathway in the viral replication is lipid metabolism that is hijacked by viruses and upregulated to meet the increased demand for viral structural elements such as the viral cell membrane [42]. Cholesterol is an important component of the viral cellular membrane. Statins reduce cholesterol synthesis by inhibiting the activity of HMG-CoA reductase. Multiple studies have eluted to the role of statins in treating different infections [55]. Tleyjeh et al. have performed a meta-analysis to investigate the role of statins in infections. They examined nine cohorts that looked at the role of statins in treating infections like bacteremia, pneumonia, and sepsis [56]. In conclusion, they showed that the adjusted effect estimate was 0.55 (95% confidence interval, 0.36-0.83; I (2) = 76.5%) in favor of statins [56]. The authors concluded that statins were effective in reducing the infection; however, due to the heterogeneity in the analyzed data, they highlighted the need for randomized clinical trials [56]. In another study, Douglas et. al., have shown that statins can protect against all caused mortality within six months of diagnosis with pneumonia with an adjusted hazard ratio of 0.67 (0.49 to 0.91) in favor of statins therapy [57]. In a randomized clinical trial, Makris et al. studied the impact of using pravastatin on reducing the frequency of ventilator-associated pneumonia. Moreover, they studied if the treatment had favorable outcomes in patients with Acute Physiology and Chronic Health Evaluation II score (ICU severity scoring classification) increasing the probability of the survival of the treated group when compared to the untreated group [58]. However, another randomized clinical trial showed no benefit for statin therapy [59]. Moreover, other studies looked at the effect of a combination therapy using atorvastatin (40 mg/day) and irbesartan (150 mg/day).

Statin’s beneficial impact on viral infection could be due to its lipid lowering effects that can potentially suppress coronavirus infection, as shown in other viruses where the cholesterol-lowering abilities disturb lipid rafts. Even though lipid-lowering capabilities might impact viral replication, statins can also help in mitigating the impact of viral infection through their immunomodulatory and anti-inflammatory properties [60]. This is particularly important since many cardiovascular patients have been shown to have an elevated COVID-19 infection risk [61]. Additionally, statins can also exert beneficial effects on COVID-19 patients through their stabilization of atherosclerotic plaques [62]. Another lipid-lowering drug that showed potential antiviral properties in vitro is fibrates [63]. Fibrates are drugs that target fatty acid synthesis and increase lipoprotein lipase activity. They have been shown to increase survival of mice infected with influenza virus [63]. Taken together, these studies suggest a beneficial impact for statins and potentially other lipid-lowering drugs such as PCSK9 inhibitors for treatment of COVID-19, especially that of the most severely infected people which are suffering from cardiovascular disease and diabetes [55].

Targeting viruses with drugs can utilize the fact that viruses lack basic metabolic processes and rather exploit their host’s metabolic machinery. Lipid metabolism is an important component of the virus life cycle as demonstrated previously; as a result, targeting the lipid metabolism pathways could constitute an early-intervention and exciting host-directed drug target. Yuan et al. utilized such a pathway and tested a lipid drug library and showed that AM580, a retinoid acid receptor alpha (RAR-a) agonist could interrupt the life cycle of MERS-CoV as well as influenza A virus [64]. It was also shown that AM580 binds to the sterol regulatory element binding protein (SREBP) in host cells. SREBPs are basic helix loop helix transcription factors that play an important role in regulating lipid metabolism which was highlighted as the mechanism of action for the antiviral activity of AM580 [64].

## 6. Sphingolipids as a Therapeutic Target

Sphingolipids are important lipids that regulate membrane properties such as viscosity and tension, which might also make them suitable as novel targets for therapeutic intervention [65,66,67]. Sphingolipid biosynthesis is modulated in so many diseases, and mutations impacting their metabolism have been shown to cause disease [65,66,67,68,69,70]. The sphingolipid biosynthesis pathway starts with the formation of 3-ketosphinganine from serine and palmitoyl-CoA by serine palmitoyltransferase (SPT). SPT is composed of two major subunits and several small subunits that are most likely involved in its regulation and specificity [71,72]. As sphingolipids, especially ceramides and glucosylceramides, accumulate in a wide variety of diseases, sphingolipid biosynthesis is widely viewed as a therapeutic target for many different indications [65,66,67].

In the past few years, it has been established that sphingolipids play a vital protective role in lungs from pulmonary leak and lung injury, and the modulation of their pathways may offer effective therapeutic intervention strategies [73]. Modulation of sphingolipids can be very beneficial and can offer an anti-inflammatory, neuroprotective, and anti-coagulant effects [74,75]. All of these exciting features could likely be exploited to counteract the associated complications of COVID-19 infection [76].

On this basis, the FDA-approved immunomodulator FTY720 (Fingolimod), one of the best characterized “sphingomimetic” drugs [75], is currently on an ongoing Clinical Trial (NCT04280588 and NCT04276688—ClinicalTrials.gov) for the management of the COVID-19 pandemic [76,77]. Fingolimod acts as a nonspecific agonist of sphingosine 1-phosphate receptors (S1PR) and as a selective functional antagonist of the S1P1 subtype by promoting receptor internalization and degradation [78]. Since S1P1 is critical in controlling lymphocyte trafficking, its downregulation causes relocation of the immune cells to secondary lymphoid tissues, causing a reduction from the circulation and henceforth immunosuppression [78]. This will reduce the central inflammation response and may help since elevated levels of inflammatory cytokines, particularly IL-6, are thought to be associated with respiratory failure in COVID-19 patients. However, it has been shown that the degree of lymphopenia is not associated with risk of infection, and generally, infectious complications were relatively low on S1P modulators [77,79]. It has also been well documented that patients taking these drugs do not suffer from an increased risk of community-acquired viral infections [77,80]. We have recently shown that a higher level of ANGPTL6 was associated with slow multiple sclerosis progression and good response to fingolimod treatment [81].

Recently, more specific functionally related compounds have been developed, that better differentiate between the various S1PR subtypes such as selective compounds KRP203, ponesimod, and cenerimod [75]. Due to their higher specificity, these new treatments hold great potential for clinical advancements in a broad range of autoimmune and inflammatory diseases. In addition to the immunomodulatory property of fingolimod, its anti-thrombotic and anti-coagulant actions [75] would make it more suitable and promising for the treatment of COVID-19 patients [76]. Nevertheless, other “sphingomimetic” and “sphingomodulating” compounds, some of which are already in clinical trials for other clinical diseases, can be easily reused and possibly provide further therapeutic options for the management of this global pandemic crisis [82].

## 7. Targeting Viral Entry Pathway

Inhibitors of virus cell entry and endocytosis as well as inhibitors of certain cellular pathways involved in the viral cycle constitute an important reservoir of antiviral drugs, such as methyl-β-cyclodextrin (MβCD), which works by depleting cholesterol [83]. In vitro cell models expressing ACE2 showed that cholesterol depletion by MβCD dramatically decreased the number of bonds with the S protein and reduced ACE2 expression in a dose-dependent manner leading to reduced SARS-CoV replication [84]. Similarly, phytosterols, which are lipophilic molecules that can interact with the lipid raft and reduce membrane cholesterol and destabilize the membrane structure, impact viral infectivity significantly. Other direct inhibitors of the endocytosis process, such as chlorpromazine, chloroquine, and umifenovir (Arbidol), have also shown promising results [48,85,86].

Another example of a broad-spectrum antiviral drug is LJ001, which is a membrane-binding compound [87,88]. LJ001 possesss a very promising antiviral mechanism that can selectively impact viral membranes but not host cellular membranes at very low antiviral doses (IC50 ≤ 0.5 µM) [87,88,89]. This compound is astoundingly capable of inhibiting a wide range of enveloped viruses including HIV, influenza, and Ebola, amongst many others [89]. Vigant et al. showed that LJ001 targets unsaturated phospholipids in a manner that was dependent on light and molecular oxygen [89]. It was postulated that LJ0001 leads to increased unsaturated fatty acid hydroxylation that primes the formation of a hydroxyl group in the middle of the hydrophobic lipid bilayer, thus impacting the viral membrane properties [89]. This drug is viral membrane-specific due to the intrinsic mechanisms in host cellular membranes that are protective against phospholipid hydroperoxides. While the effects of LJ0001 are limited to in vitro activity, since it is a light activated compound, newer compounds such as oxazolidine-2,4-dithiones with membrane-intercalating photosensitizers were designed. These examples clearly highlight the importance of the viral membrane as antiviral targets [89].

## 8. Conclusion and Future Direction

Lipid metabolism plays an important role in the viral infection cycle. This role can be harnessed as anti-viral drugs that can constitute a broad-spectrum drug that can be utilized in a makeshift fashion that offers a first response drug. The best example of such drugs are statins that has shown promising beneficial effect in people with various viral infections alongside their main role as a lipid lowering therapy. As a result, more work is needed to focus on the role played by lipid species at the structure and signaling pathway level in the viral life cycle. This will allow us to establish new drug targets as well as enhance existing drugs. Such lipid-based therapies can be used alone or in combination with other drugs, but they will have the advantage of targeting multiple viruses.
